# Deciphering Hyperammonia-Producing Bacteria (HAB) in the Rumen of Water Buffaloes (*Bubalus bubalis*) and Their Inhibition through Plant Extracts and Essential Oils

**DOI:** 10.3390/microorganisms12102040

**Published:** 2024-10-09

**Authors:** Yendrembam Mery Chanu, Shyam Sundar Paul, Avijit Dey, Jerome Andonissamy

**Affiliations:** 1 Division of Animal Nutrition and Feed Technologies, ICAR-Central Institute for Research on Buffaloes, Hisar 125001, Haryana, India; 2 Division of Animal Physiology & Reproduction, ICAR-Central Institute for Research on Buffaloes, Hisar 125001, Haryana, India

**Keywords:** buffalo, hyperammonia-producing bacteria, rumen, plant bioactive compounds

## Abstract

Hyperammonia-producing bacteria (HAB) are a class of microbes present in the stomach of ruminants, responsible for the rapid rate of ammonia production from protein degradation beyond the capacity of these animals for their utilization. Thus, ruminant nutritionists are interested in decreasing ruminal protein degradation and ammonia genesis by focusing on controlling the activity of HAB. The investigations of the present study were carried out to determine predominant hyperammonia-producing bacteria in the rumen of buffaloes, their isolation and characterization, as well as the inhibition of these isolates with various sources of plant secondary compounds (tannins, saponins, and essential oils). Studies employing high-throughput sequencing of amplicons of the 16S rRNA gene from genomic DNA recovered from enrichment culture of HAB of buffalo rumina indicated that, at the phylum level, *Proteobacteria* (61.1 to 68.2%) was the most predominant HAB. *Acidaminococcus* was most predominant among the identified genera. In vitro experiments were conducted with enrichment culture of buffalo rumen contents incubated with different types of feed additives such as essential oils (eucalyptus oil, lemon grass oil, and clove oil) and extracts of plants (*Sapindus mukorossi* fruits and *Ficus bengalensis* leaves), each at graded dose levels. The reduction in ammonia production by clove and lemon grass oils was evident due to the presence of major bioactive compounds, especially eugenol and limonene, which have strong antimicrobial activity. However, clove oil and Indian soapberry/reetha (*Sapindus mukorossi)* fruit were found to be promising and effective in reducing the growth, protease production, and ammonia production of HAB culture.

## 1. Introduction

In buffaloes, like other ruminants, feedstuffs are subjected to fermentation in the rumen before intestinal degradation. This fermentation gives them the ability to digest cellulose, but some products of digestion (ammonia and methane) are deleterious. Ruminal amino acid deamination deprives the animal of amino acids [1]. Most of the amino acid nitrogen that enters the rumen (60–90%) is deaminated, and the rates of ammonia production often exceed the needs of ammonia-utilizing microorganisms [2,3,4]. This results in the excess absorption of ammonia across the rumen wall and conversion into urea and excretion through urine and feces. Only 5 to 30% of nitrogen from animal feed is retained and the rest is excreted. Therefore, excessive protein degradation and amino acid deamination in the rumen represents a loss of dietary protein for the animal because it is a nutritionally wasteful process [5,6]. Ruminant nitrogen excretion is a major source of environmental pollution [7], and nitrogen excretion by livestock and nitrous oxide emissions from manure pose major concerns, as both contribute to air and groundwater pollution and have been associated with an increased risk of pulmonary disease [8]. Hence, nutritionists have attempted to combat this excess ruminal nitrogen degradation using proteins that are naturally insoluble and/or using heat and formaldehyde treatments that decrease solubility. However, these approaches increase the cost of the ration significantly [9,10]. Also, the use of ionophore viz. monensin can also decrease amino acid degradation [11], but there has been increasing concerns over the use of antibiotics in animal feed [12]. 

The buffalo (*Bubalus bubalis*) is a vital animal in Asian countries, providing milk, meat, and draught power, leading towards socioeconomic upliftment, and ensuring the livelihood of the people of the region [13]. With more than half of the world’s buffalo population, India is the leader in global milk production and the exportation of buffalo meat. Like other ruminants, buffalo rumen microbes are of a diverse nature; however, their population, composition, and functional properties vary with feeding habits. The excessive degradation of protein in buffalo rumens and excessive generation of ammonia, beyond the capacity to produce microbial protein, results in dietary and economic loss [14].

Thus, ruminant nutritionists have recently increased their focus on decreasing ruminal protein degradation and ammonia genesis by focusing on controlling the activity of hyperammonia-producing bacteria [5,15], because the hyperammonia-producing bacteria (HAB) are bacteria that can produce ammonia at a rapid rate (>300 nmol NH_3_/mg cell protein/min). HAB have minimal ability to utilize sugars [16,17], and a majority of them are considered to form an amino acid-utilizing niche; however, a few HAB can utilize peptides. Although only present in low numbers inside the rumen, the concentration of ammonia they can produce makes them quantitatively important. HAB have been isolated in cattle, sheep, and goats [18].

Many researchers have proposed lowering methane and ammonia production in the rumen using plant secondary metabolites. Through secondary metabolism, many plant species produce a variety of organic compounds. These secondary metabolites are often placed into one of three groups: saponins, tannins, and essential oils; some exhibit antimicrobial activity against many bacteria, yeasts, and even molds [19]. Natural plant products containing essential oils (EOs), saponins, or tannins have been widely evaluated as non-antibiotic feed additives to mitigate methane emission, suppress protein degradation in the rumen, increase feed utilization efficiency, and improve the characteristics of rumen fermentation [20,21].

However, these plant bioactive compounds (PBACs) are specific in terms of their ability to inhibit a category of microbes. As rumen microbial community structures are known to be dependent on host species, the effectiveness of PBACs depends on their concentrations as well as the species of animal. There is very scant information available on HAB present in the rumen of buffaloes and PBACs, which are effective against such bacteria. Considering all these aspects, the purpose of this study was to determine the predominant genera of hyperammonia-producing bacteria in the rumen of buffaloes, their isolation and characterization, as well as the inhibition of these isolates with various plant secondary compounds.

## 2. Materials and Methods

The experiment was conducted at the Division of Animal Nutrition and Feed Technologies, ICAR-Central Institute for Research on Buffaloes (CIRB), Hisar, Haryana, India (29.1203_N, 75.8069_E). The approval of the Institutional Animal Ethics Committee (IAEC-CIRB/16-17/A/09, dated 15 July 2016) for the care of rumen-fistulated animals and for rumen fluid collection was obtained for the experiment.

### 2.1. Determination of Predominant HAB in Rumen of Buffalo

Enrichment culture of rumen fluid of 4 buffaloes was prepared in semidefined medium with trypticase or casamino acid as the sole carbon source. After stabilization (6 transfers), genomic DNA was isolated from the enrichment cultures as described below and subjected to high-throughput sequencing of the 16S rRNA gene using an Illumina MiSeq sequencing platform in 2 × 300 bp mode and universal primers as per standard protocol recommended by manufacturer. The data were subjected to bioinformatic analysis using the MOTHUR pipeline [22] to determine the predominant genera of microbes present in these enrichment cultures. 

#### 2.1.1. Preparation of Enrichment Medium

The bacterial enrichment medium was prepared as per the composition described by Russell et al. [23]. All of the components excluding cysteine-HCl and sodium sulfide (Na_2_S × 9H_2_O) were taken in a conical flask and heated over a flame to reduce the oxygen content until the solution became colorless. Then cysteine-HCl and sodium sulfide were added to the liquid medium slowly and the medium was heated for 1–2 min and immediately bubbled with CO_2_ to make it saturated with CO_2_. After 15–20 min of gassing, the medium was dispensed into Hungate’s tubes with a 5 mL syringe. The Hungate tubes containing medium were then autoclaved at 20 psi for 20 min and stored at room temperature for further use.

#### 2.1.2. Enrichment of Hyperammonia-Producing Bacteria (HAB)

Fresh rumen fluid was collected before morning feeding from four rumen cannulated Murrah buffalo steers (avg. age 2.5 years, 380 ± 14 kg B.W.) fed on a basal diet of wheat straw offered ad libitum and a limited amount of standard concentrate mixture (19% crude protein and 72% total digestible nutrients) and green oat fodder in the morning (09:30 h) to meet their nutrient requirements for maintenance [24]. Clean drinking water was provided freely to the animals housed in the well-ventilated sheds with individual feeding. Rumen content was collected before feeding and watering the animals between 09:00 and 09:30 h according to standard procedures. An equal volume of rumen content from each animal was pooled and brought to the laboratory in a pre-warmed thermos flask for further use. All laboratory handling of rumen content was conducted under continuous flushing with CO_2_ to maintain anaerobic conditions. 

A total of 5 g of the rumen content was directly put into Hungate’s roll tubes containing liquid enrichment medium [23] and placed into an incubator at 39 °C for 48 h. After 2 days, bacteria growth was observed, and it was transferred to sterile tubes containing liquid medium. Similarly, after several transfers (6–9 transfers), the culture was considered stabilized and enriched with HAB and stored at 4 °C for further use.

#### 2.1.3. Isolation and Characterization of HAB

From enrichment cultures, samples were drawn and serially diluted and the isolation of bacterial colonies was carried out in agar roll tubes containing casamino acid/trypticase as sole substrate. Twenty isolates were obtained. Colonies were transferred to enrichment media broth [23] and repeated 4 times or until morphologically pure colonies were obtained. Isolates were evaluated for growth rate (by measuring OD at 0, 6, 12, and 24 h in liquid culture) and rate of ammonia production by colorimetric method, protease assay, morphology, including Gram staining, and also by PCR amplification of the 16S rRNA gene followed by sequencing and bioinformatic analysis. The isolates having the fastest rate of ammonia production were characterized by their ability to utilize different substrates through biochemical tests viz. protein assay and ammonia nitrogen tests.

##### Preparation of Solid Medium

The composition of the solid medium was similar to that given for the liquid medium with the additional component of 2% agar. We weighed agar (60 mg for 3 mL medium per roll tube) in each roll tube and gassed for 5–6 min to flush out O_2_ present in agar and 3 mL of liquid medium was dispensed into it. The roll tubes were sealed and autoclaved. The tubes were stored at room temperature until further processing.

##### Anaerobic Dilution Medium

The composition of anaerobic dilution medium [25] is as follows: mineral solution I, (15 mL); mineral solution II, (15 mL); 0.1% (*w*/*v*) resazurin (0.1 mL); sodium bicarbonate (0.4 g); distilled water, 70 mL. Mineral solution I consists of K_2_HPO_4_ (3 g/L of distilled water) and mineral solution II per 1000 mL of distilled water consists of NaCl (6 g); NH_4_Cl (1.25 g); KH_2_PO_4_ (3 g); CaCl_2_ × 2H_2_O (0.8 g); MgCl_2_ × 2H_2_O (0.6 g). The mixture was boiled for 4–5 min to reduce oxygen content and then cysteine-HCl (60 mg) and sodium sulfide (30 mg) were added. CO_2_ was bubbled until the mixture became colorless. A total of 9 ml of this medium was dispensed in each of the Hungate’s roll tubes and autoclaved at 20 psi for 20 min.

##### Isolation of HAB Bacteria

Rumen fluid of fistulated buffaloes was collected in a serum bottle which was previously flushed with CO_2_ to maintain an anaerobic condition, and after collection, the tube was brought to the laboratory immediately and the rumen content of 5 g was taken from the tube and directly put into a Hungate’s roll tube containing liquid medium, and this was placed in an incubator for 24 h. The next day, bacteria growth was observed; if growth was less, then incubation was continued until and unless optimal growth was found. From enrichment cultures, samples were drawn and serially diluted (10^−8^) and isolation of bacterial colonies was carried out in agar roll tubes containing casamino acid/trypticase as sole substrate. The bacterial colonies were observed after 3–6 days and isolated colonies were transferred to broth and the process was repeated 4 times or until morphologically pure colonies were obtained. Twenty pure culture isolates were obtained. The purity was verified under a microscope based on morphology. The pure culture in Hungate’s tubes was stabbed in agar roll tubes, allowed to grow overnight, and then stored at 4 °C for medium-term storage/preservation. 

##### Identification of Anaerobic Bacteria

Identification of the form of bacterial cell was carried out based on morphological features including Gram staining using a *Motic B series* compound microscope (Vancouver, BC, Canada) at 100× magnification.

##### Estimation of Ammonia Production by Colorimetric Method

Ammonia production (nmol NH_3_/mg cell protein/mL) was estimated by a colorimetric method based on phenol-hypochlorite reaction using ammonium sulfate standard solution as described by Weatherburn [26].

##### Estimation of Protein

Estimation of protein was carried out using Quick Start ^TM^ Bradford protein assay [27]. This assay makes it possible to quickly assay multiple protein samples while using a small sample volume (5 μL). It is also possible to automate protein determination with this multiwell plate assay (96-well plate). The absorbance of the protein–dye complex was determined at 595 nm using a BSA protein standard.

##### Estimation of Protease by Azocasein Method

Estimation of protease by Azocasein method was carried out as described previously [28].

Estimation of the enzyme activity was carried out as follows: enzyme activity (mIU/mg cell protein/mL) = μg hydrolyzed azocasein/h/mL = µg hydrolyzed azocasein/(Time × Sample volume).

##### Evaluation of Growth Rate in Liquid Culture

The evaluation of the growth rate of the highest ammonia-producing isolate among the 20 isolated pure cultures was carried out at different hours (0, 6, 12, and 24 h) in liquid culture by measuring optical density at 600 nm in a spectrophotometer. 

##### Isolation of Bacterial DNA

Bacterial DNA was extracted from the pure culture medium by the GES method, as described previously by Pitcher et al. [29]. DNA was extracted from the enrichment cultures following the bead beating plus column method described by Yu and Morrison (2004) using the DNA purification columns from the QIAamp Fast DNA Stool Mini kit (QIAGEN, Hilden, Germany). DNA concentration and quality were assessed using a Qubit 3.0 fluorometer (ThermoFisher Scientific, Waltham, MA, USA; #Q33238) and DNA HS Assay Kit (Thermofisher, Waltham, MA, USA; #Q32851) and also by gel electrophoresis. DNA was stored in a freezer at −20 °C until further processing.

##### Polymerase Chain Reaction (PCR) Amplification of 16S rRNA Gene

The composition of PCR amplification (16S rRNA gene) reaction mixture for identification of HAB (used for sequencing) isolates has been presented in Table 1, and the primer set used for PCR amplification has been presented in Table 2.

The PCR started with 5 min at 95 °C for Taq polymerase activation followed by 30 cycles of denaturation at 94 °C for 1 min, annealing at 55 °C for 30 s, and elongation at 72 °C for 1 min 30 s. The reactions were ended with a final elongation step at 72 °C for 7 min.

The amplified PCR products were purified using a Qiagen PCR purification kit and subjected to Sanger sequencing. DNA sequences were quality-checked and filtered using FastQC and MOTHUR software. Sequences were taxonomically classified using the RDP classifier and subjected to BLAST search in GenBank.

##### Substrate Utilization by Bacterial Isolates

The bacterial isolate having the highest rate of ammonia production was selected and it was further characterized for its ability to grow on different amino acids, viz. lysine, methionine, phenylalanine, leucine, isoleucine, threonine, histidine, arginine, tryptophan, and valine. After 2 days of incubation, measurements were conducted for growth rate, ammonia production, and protease assay. These processes were repeated until the stabilization of the culture in different substrate amino acids viz. lysine, methionine, isoleucine, threonine, and arginine (4–5 transfers). 

### 2.2. Evaluation of Additives for Inhibition of HAB

The extracts of plant secondary metabolites [*Sapindus mukorossi* ethanol (SME) and acetone extracts (SMA), *Ficus bengalensis* acetone (FBAC), and aqueous extracts (FBA)] were prepared at the laboratory [31] and examined at a graded level (6, 12, 18 mL/L); however, pure essential oils (eucalyptus oil, lemongrass oil, and clove oil), procured from Sigma Aldrich Chemicals Pvt. Ltd., New Delhi, India, were added at graded dose levels (0.4, 0.5, 0.6 mL/L) directly into Hungate’s roll tube containing liquid medium (5 mL) under CO_2_ flash. After closing the septum and cap, these tubes were mixed (gentle vortex) properly and inoculated with 1% (*v*/*v*) of mixed rumen HAB enrichment culture. These tubes were incubated for different amounts of time (0, 12, 24 h) to evaluate the growth rate, ammonia production, and protease assay. The best plant secondary metabolites and essential oils having the highest inhibition of growth/ammonia production/protease production in HAB enrichment culture were selected for in vitro trials. Screening of plant extract was also carried out in a similar way as described for plant secondary metabolites and essential oils.

### 2.3. Amplicon Sequencing and Analysis

Enrichment cultures of HABs were characterized by sequencing the v3-v4 region of the 16S rRNA gene for 4 DNA samples. Extracted DNA was PCR-amplified using the primer pair Ba27F (5′-GAGTTTGATCMTGGCTCAG-3′) and Ba515Rmod1 (5′-CCGCGGCKGCTGGCAC-3′) [32]. PCR amplification was performed using Kapa Hi-Fi Hot Start master mix for each amplicon separately for each sample. The amplified products were purified using Ampure beads and quantitated using Qubit followed by library preparation. Library preparation using the Nextera DNA library kit (Illumina Inc, San Diego, CA 92121, USA). The quantification and quality estimation of the library was carried out in an Agilent 2200 Tape Station. Sequencing was performed using an Illumina MiSeq sequencer in 2 × 300 bp pair-end sequencing mode. Three negative controls per plate were also placed in the sequencing run to detect spurious amplification. The raw reads were demultiplexed using Miseq Reporter software (version 2.4) and the bcl2fastq2 tool (version 2.2) of Illumina and evaluated using FastQC (version 0.11.8). Reads with a Phred quality score (Q) lower than 20 were removed during downstream processing. Primers and adapters were trimmed using in-house Perl scripts. MOTHUR software package (V 1.47.0) [22] was utilized for further quality screening, assembly of reads, alignment against SILVA [33] seed alignment (v 138), trimming of alignment, denoising using pre.cluster algorithm, chimera removal using UCHIME algorithm [34], and taxonomic classification using RDP classifier against RDP classifier training set v 19 [35]. 

### 2.4. Statistical Analysis

All the biochemical analyses were performed in duplicates. The data on in vitro fermentation parameters in enrichment cultures were analyzed by one-way analysis of variance using SPSS, (version 17.0) [36]. Specific differences between pairs of means were tested using Duncan’s multiple range test at *p* < 0.05. Differences between treatment means were considered significant when *p* < 0.05 and a trend was considered to exist when 0.05 < *p* < 0.10.

## 3. Results

### 3.1. Predominant Hyperammonia-Producing Bacteria in the Rumen of Buffaloes

The rumen contains a diverse population of bacteria and isolation of their pure cultures and their characterization has limitations in terms of the number of isolates that can be handled. Enrichment culture is a kind of culture method that aims at increasing the proportion of the target bacteria, tending to a pure culture. Enrichment culture is mainly used to control physiological factors such as C source, N source, pH, temperature, oxygen demand, etc., of the culture medium, and repeated transfer of culture in such a medium tends to eliminate nontarget microbes. In the present study, buffalo rumen content was subjected to enrichment culture in a medium containing casamino acid as the sole C source, which is expected to favor a population of HAB selectively. After the fourth passage of the enrichment culture, the metagenomic DNA was isolated and used as a PCR template to amplify 16S rRNA genes of bacteria, and the amplicons were used for high-throughput MiSeq sequencing. The quality-checked sequence reads were subjected to taxonomic classification using an RDP Classifier to assess the detail diversity of HAB in the rumen of buffalo. In total, 344630, 752801, 689007, and 310815 quality-checked reads of bacterial 16S rRNA gene were obtained in fistulated animals 1, 2, 3, and 4, respectively (Table 3). The reads were classified as per RDP into major taxonomic groups (having at least 1000 reads in at least one animal) including seven phyla, nine families, and eleven genera. At the phylum level, *Proteobacteria* (61.1- 68.2%) was found to be the most predominant, followed by *Unclassified bacteria* (24- 29%), *Firmicutes* (2.8–6.9%), *Bacteroidetes* (0.9–2%), *Actinobacteria* (0.1–1.1%), *Fusobacteria* (0.04–0.7%) and *Synergistetes* (0.1–0.4%). At the family and genus level, most of the sequences remained unclassified. Among sequences with a valid family name, *Acidaminococcaceae* was the most predominant, constituting 1.5 to 4.7% of sequences. Similarly, at the genus level, among sequences with a valid genus name, *Acidaminococcus* was the most predominant, constituting 1.4–4.1% of total bacterial sequences.

### 3.2. Results of Isolation and Characterization of Hyperammonia-Producing Bacteria (HAB)

In this study, 20 pure bacterial isolates were isolated from an enrichment culture of buffalo rumen fluid in a medium containing casamino acid as the sole energy source and the isolates were characterized by the Gram staining method. All 20 isolates were subjected to the determination of ammonia production by colorimetric method at 0 h, 6 h, and 24 h. Further, protease assay was also undertaken at 6 h and 24 h. The average growth rate of the highest ammonia-producing isolate among the 20 isolates was also determined at 0, 6, 12, and 24 h intervals. The highest hyperammonia-producing bacterial (HAB) isolate was selected, and it was further characterized for its ability to utilize different amino acids (histidine, methionine, threonine, lysine, leucine, isoleucine, phenylalanine, arginine, and valine) as a substrate. 

The data on morphological characteristics of HAB bacteria are presented in Table 4. The isolates were Gram-positive cocci (HAB1, HAB3, HAB5, HAB16, HAB17), Gram-positive rod (HAB2, HAB7, HAB8, HAB12, HAB13, HAB14, HAB15), Gram-negative cocci (HAB4) and Gram-negative rod (HAB6, HAB9, HAB10, HAB11, HAB18, HAB19 and HAB20). The data on ammonia concentration in culture medium of HAB isolates at 0 h, 6 h, and 24 h of incubation are presented in Table 5. In general, the ammonia concentration at 24 h post-incubation was much higher than that of 6 h of incubation. At 0 h of incubation, the range of ammonia concentration in different cultures of HAB was 0.10 to 0.97 mg N/dL. At 6 h of incubation, the range of ammonia concentration was 17.0 to 118.1 mg N/dL. At 24 h of incubation, ammonia concentration ranged from 0.9 to 167.8 mg N/dL.

The data on ammonia production rate (Table 5) in terms of nmol NH_3_/mg cell protein/min by different HAB isolates demonstrated that the rate of ammonia production at 6 h post-incubation was much higher than that at 24 h. The rate of production of protease enzyme (Table 6) by different isolates indicated an increased protease production at 24 h post-incubation than that of 6 h and variation between isolates was very low. The data on the rate of growth (measured by OD value of culture medium) of the top 8 isolates of HAB are presented in Table 7. There was a gradual increase in OD values with the passage of time and variations between isolates were low.

### 3.3. Amino Acid Utilization Ability of the Best HAB Isolate

The best hyperammonia-producing bacteria among 20 HAB isolates (HAB 14) was cultured in a medium containing different amino acids as the sole carbon source. The growth was assessed by ammonia and protease production up to four transfers. The data on ammonia production on different amino acids are presented in Table 8. Among the different amino acids (histidine, methionine, threonine, lysine, leucine, isoleucine, phenylalanine, arginine, and valine), the least ammonia production was observed in the medium containing histidine as the sole carbon source as compared to the control (casamino acid).

Data on protease production in the culture medium containing different amino acids as the sole carbon source by the best HAB isolate are presented in Table 9. Among the different substrates (histidine, methionine, threonine, lysine, leucine, isoleucine, phenylalanine, arginine, and valine), the least protease production was observed in histidine as compared to control.

The growth rate of the best HAB isolates (optical density or absorbance values of culture medium at 600 nm wavelength) at 48 h of incubation in a medium containing different amino acids as the sole carbon source has been presented in Table 10. Among the different substrates (histidine, methionine, threonine, lysine, leucine, isoleucine, phenylalanine, arginine, and valine), the best growth was noticed in arginine.

### 3.4. 16S rRNA Gene Sequence-Based Identification of HAB Isolates

The result of BLAST (basic local alignment and search tool) search of 16S rRNA gene sequences of the highest ammonia-producing isolates in GenBank database has been presented in Table 11 and demonstrated nine superior isolates of HAB to the nearest database.

### 3.5. Evaluation of Plant Bioactive Compounds for Inhibition of HAB

A series of in vitro experiments were conducted where enrichment culture of buffalo rumen contents was incubated with different types of feed additives such as essential oils (eucalyptus oil, lemongrass oil, and clove oil) and extracts of plants (*Sapindus mukorossi* fruits and *Ficus bengalensis* leaves), each at graded dose levels, and suppression of ammonia production was used as an indicator of inhibition of deaminase activity of HAB. Protease production and growth rate in terms of OD at 600 nm of culture medium were also recorded.

The first set of experiments was conducted to evaluate the effects of three graded levels of eucalyptus oil (eucalyptol), lemongrass oil, and clove oil on ammonia concentration in an enrichment culture medium (Table 12). The results revealed that, at 12 h of incubation, the ammonia production was significantly (*p* < 0.01) decreased in lemongrass oil- and clove oil-supplemented groups, irrespective of dose levels; however, no effects (*p* > 0.05) were evident in eucalyptus oil supplementation, in comparison to the non-supplemented control. However, at 24 h post-incubation, only clove oils, irrespective of dose levels, and lemongrass oils at the highest dose (0.6 mL/L) continued to suppress (*p* < 0.001) ammonia production. The protease concentration in culture medium revealed (Table 10) no significant difference (*p* > 0.05) at 12 h between control and treatments, while at 24 h, only clove oil at the highest dose (0.6 mL/L) demonstrated a significant reduction (*p* < 0.01) in protease level.

The findings on the effect of different doses of eucalyptus oil, lemongrass oil and clove oil on the growth of HAB enrichment culture revealed (Table 13) no significant effects of the treatments except for the higher (*p* < 0.01) doses (0.5–0.6 mL/L) of clove oil.

The second set of experiments was conducted to examine the effects of extracts of *Sapindus mukorossi* (Reetha) fruits (ethanol and aqueous extract) and *Ficus bengalensis* (Bargad) leaves (acetone and aqueous extract) at different dose levels, and the results on ammonia concentration in culture medium indicated (Table 14) that water extract of *Sapindus mukorossi* fruits reduced (*p* < 0.01) ammonia concentration in HAB enrichment culture both at 12 and 24 h, but ethanol extract was effective (*p* < 0.01) at 24 h only in comparison to the respective solvent control. The aqueous extract of *Ficus bengalensis* leaves (FBA) was ineffective (*p* > 0.05) in reducing ammonia production by mixed rumen bacteria in enrichment culture at 12 h incubation; however, a reduction was evident (*p* < 0.01) at 24 h. The acetone extract of *Ficus bengalensis* leaves (FBAC) was effective in reducing (*p* < 0.01) ammonia production at both 12 and 24 h at both dose levels (12 and 18 mL/L) in comparison to the respective control with acetone solvent; however, the lowest dose level examined (6 mL/L) remained ineffective.

*Ficus bengalensis* leaf extracts (acetone as well as aqueous extract) and aqueous extracts of *Sapindus* had a negligible effect (*p* > 0.05) on protease level at both 12 and 24 h, but ethanol extract of *Sapindus* had higher (*p* < 0.05) protease activity at 12 h, but lower activity at 24 h (Table 14). The growth rate of the culture medium of HAB upon the addition of extracts of *Ficus* and *Sapindus* demonstrated a reduction at both 12 and 24 h with ethanol extract of *Sapindus*; however, aqueous extract of *Sapindus* was effective only at 12 h incubation. The acetone extract of *Ficus bengalensis* leaves was effective in reducing the growth of HAB at 12 h only, irrespective of levels; however, the highest dose (18 mL/L) also reduced (*p* < 0.01) the growth at 24 h also, although the aqueous extract was ineffective (*p* > 0.05) at both 12 and 24 h, irrespective of dose levels (Table 15).

## 4. Discussion

### 4.1. Determination of Predominant Hyperammonia-Producing Bacteria (HAB) in the Rumen of Buffaloes

Ruminant animals obtain most of their nutrients from fermentation products produced by the ruminal microbiome consisting of bacteria, archaea, protozoa, and fungi. Although many studies have been reported that characterize the ruminal microbiome using cultivation-based methods, the isolated species accounted for only a small proportion of the rumen microbiome [37,38]. In the ruminal microbiome, bacteria were the most abundant domain and greatly contributed to the production of the fermentation products. Bacteria capable of utilizing trypticase/casamino acid, producing ammonia at a rapid rate, and with constrained ability to derive ATP from sugar fermentation, became known as hyperammonia-producing bacteria (HAB). Although their number in the rumen is meager, they constitute an important functional group in the rumen and contribute to the inefficiency of protein utilization and yet remain poorly characterized so far. Earlier, cloning and sequencing of 16S rRNA genes were used to identify ruminal microorganisms in primarily domesticated ruminant animals, but also in wild ruminant animals [39,40]. Because of the limited number of clones sequenced in individual studies, only predominant members of the ruminal microbiome were identified. To overcome these limitations, in the present study, metagenomic DNA isolated from an enrichment culture of mixed microflora of buffalo rumen content in a medium favoring the growth of HAB was analyzed by high-throughput next-generation sequencing using the MiSeq sequencing platform. This sequencing-by-synthesis technology allows for the analysis of sequences of 16S rRNA genes of thousands of microbes without requiring cloning of genes of individual microbes or their culturing. This allowed us to examine the diversity of the ruminal microbiome in HAB enrichment culture. At the phylum level, the sequence reads were represented mainly by the proteobacteria phylum (61–68%) followed by *Unclassified bacteria* (24–29%), *Firmicutes* (2.8–6.9%), *Bacteriodetes* (0.9–2%), *Actinobacteria* (0.1–1.1%), *Fusobacteria* (0.04–0.7%) and *Synergistetes* (0.1–0.4%). At the family and genus level, most of the sequences remained unclassified. Among sequences with a valid family name, *Acidaminococcaceae* was the most predominant, constituting 1.5 to 4.7% of sequences. Similarly, at the genus level, among sequences with a valid genus name, *Acidaminococcus* was the most predominant, constituting 1.4 to 4.1% of total bacterial sequences. At the family level, *Enterobacteriaceae*, *Bacteroidaceae*, *Ruminococcacae*, *Veillonellacae*, unclassified families under the *Clostridiales* order, *Coriobacteriaceae*, *Synergistaceae*, and *Fusobacteriaceae* were detected. At the genus level, *Candidatus Carsonella*, *Allisonella*, *Oscillobacter*, *Bacteroides*, *Proteus*, *Fusobacterium*, *Pyramidobacter*, *Paraeggerthella*, *Denitrobacterium*, *Succinoclasticum* and *Acidaminococcus* were recovered.

We could not find any other report on high-throughput culture-independent analysis of the diversity of HAB of the rumen, and no study could even be outlined on the characterization of HAB by isolation from the rumen of buffalo; hence, a direct comparison of our result with previous data could not be made. 

The known HAB in the rumen include *Clostridium sticklandii*, *C. aminophilum*, *Peptostreptococcus anaerobius, Proteus mirabilis, Fusobacterium ulcerans* and *Bacillus sp.* [41]. However, HAB species may vary between ruminants. Krause and Russel [42] were able to detect *C. sticklandii*, *C. aminophilum*, and *P. anaerobius* in cattle, whereas Attwood et al. [43] demonstrated *P. anaerobius*, but not *C. aminophilum* and *C. sticklandii*, in New Zealand sheep. Gano [15] described *Peptostreptococcus anaerobius, Proteus mirabilis, Fusobacterium ulcerans* and *Bacillus sp.* in Jersey cows. In other studies, several other novel bacteria have been identified with rapid rates of ammonia production that are taxonomically different from the known isolates [44,45], suggesting that many HABs remain uncultured. 

Some of the sequences of known HAB, as reported in earlier studies, were also recovered in the present study, but in the present study, many more additional groups were identified. Most of these sequences could not be classified down to the family or genus level owing to their low similarity with known bacterial sequences in the taxonomy database. This indicated that much a higher diversity of HAB might exist in the rumen of buffalo than known so far.

### 4.2. Isolation and Characterization of Hyperammonia-Producing Bacteria (HAB)

Twenty isolates of HAB were found to produce ammonia at a high rate (up to about 200 times higher than that reported by Gano [15] in a Casamino acid medium). Morphologically, the majority of isolates were Gram (−) rod (8) followed by Gram (+) rod (5) or Gram (+) cocci and Gram (−) cocci (2). Some of the top HAB isolates (nine isolates) were identified by sequencing of 16S rRNA gene. The top isolate (HAB 14) had the highest sequence similarity to *E. coli* among cultured bacterial sequences available in the GenBank database but the percent similarity was only 95%, indicating a potentially different and novel genus. Similarly, HAB2, HAB17, HAB 18 had <95% similarity to known bacterial genera, also indicating potential novel genera. These isolates suggest the existence of additional culturable HAB bacterial genera or species beyond the known HAB reported so far. 

The ammonia-producing bacteria isolated from the rumen had low specific activities of ammonia production when they were compared with those from mixed cultures because many ruminal bacteria are unable to produce any ammonia [46]. Therefore, specific media for enrichment bacterial cultures are necessary to examine ammonia production as well as to isolate pure cultures. Pittman and Brayant [47] reported that *P. ruminicola* 23 could not grow well in the medium containing NH_4_Cl as the only nitrogen source but Han and Jih [48] reported that the growth performance of *P. ruminicola* 23 in medium containing 0.5% of various carbon sources and 0.2% of various nitrogen sources was slow, but when they used peptone and 0.1% NH_4_Cl as nitrogen source and 0.5% sucrose as carbon source, they observed the best growth. Russell et al. [23] reported that a rate of production of 3.9 nmol NH_3_/mg protein/min was observed when strained ruminal digesta were incubated with trypticase, but a rate five times higher was observed when a batch culture type of incubation of diluted ruminal fluid was incubated with 15 mg/mL trypticase in fresh medium. McSweeney et al. [44] isolated a group of bacteria from the rumen fluid of sheep and goats fed tannin-rich (*Calliandra calothyrsus)* diets that could grow rapidly on peptides and amino acids and had proteolytic activity. Many researchers [2,17,41] reported that selected HAB isolates had a specific activity of ammonia production ≥ 100 nmol NH_3_/mg protein/min, and these bacteria were highly active in amino acid deamination. The HAB isolates obtained from the rumen of cattle, sheep, and deer could ferment different carbohydrates, including glucose, fructose, cellobiose, xylose, maltose, and trehalose, but the deamination rates of these isolates were not determined [41].

In a study, Chen and Russell [2] showed that when *Peptostreptococcus* was grown in a medium containing only serine, leucine, phenylalanine, and tyrosine, the growth rate was less than 10% when observed with casamino acid. They also observed that ruminal *Peptostreptococcus* was able to grow rapidly on the peptide source trypticase but less than 23% of the nitrogen was converted to ammonia or cell protein; however, when casamino acids were substituted for trypticase, approximately 40% more ammonia was produced. These results may be due to extracellular peptidase activity. Russell and Strobel [49] reported that *Fusobacterium necrophorum*, a Gram (−), nonmotile, and rod-shaped bacterium, degraded lysine with a very high rate of deamination (2400 nmol NH_3_/mg protein/min). We have studied the amino acid utilization pattern for only the top HAB isolate (HAB 14) and amino acid utilization data (ammonia production and change in OD) indicated only the utilization of arginine and to some extent leucine by the top HAB isolate (HAB 14). Deamination of amino acids in the rumen leads to the loss of NH_3_ across the ruminal wall and is one of the main causes of inefficient nitrogen utilization by ruminants [50] and excessive NH_3_ production in the rumen is a major nutritional inefficiency in ruminant animals. Through further isolation and characterization, a new strategy can hopefully be developed for reducing nitrogen output from buffalo.

### 4.3. Evaluation of Plant Bioactive Compounds for Inhibition of HAB

Ammonia genesis in the rumen is a wasteful process. Amino acid-fermenting bacteria, particularly hyperammonia-producing bacteria (HAB), can deaminate amino acids quickly within the rumen, causing excess ammonia production. Recently, emphasis has been given to limiting the use of antibiotics in animal feed for the fear of the development of antibiotic resistance and consequent health risks to the human population. Various plant bioactive compounds are being utilized as an alternative to antibiotics to control the activity or growth of specific groups of bacteria. In line with these developments, plant bioactive compounds are being contemplated as potential agents for modulating rumen function, including ammonia production. These secondary metabolites primarily interact with the bacterial cell membrane and have been explored for their ability to mitigate ammonia genesis in the rumen using either single species of HAB or a mixed culture of rumen microbial communities. 

Buffalo rumen content was subjected to enrichment in a medium containing casamino acid as the sole carbon source, which is expected to favor the growth of HAB. The enrichment culture was dosed with different plant bioactive compounds, and the change in ammonia concentration in the medium was monitored at 0, 12, and 24 h as an indicator of deaminase activity. Furthermore, protease level and growth of HAB (OD of culture medium) were also assessed. The reduction in ammonia production by clove and lemon grass oils, in the present study, could be due to the presence of many bioactive compounds, especially eugenol and limonene, respectively, which have strong antimicrobial activity. In support of our study, Gano [15] showed that clove oil, when used at a dose of 0.5 g/L, decreased ammonia concentrations in the enriched HAB culture at both 12 h and 24 h post-incubation. Busquet et al. [51] also reported that clove oil inhibited ammonia from mixed ruminal cultures in vitro, owing to its eugenol content, which contributes to the antimicrobial capabilities of clove oil. As a phenylpropanoid, eugenol contains an aromatic ring of six carbons. Similar to terpenes, eugenol is capable of penetrating the lipopolysaccharide layer, altering cell structure, and causing leakage of the cell membrane. Limonene and citral, the main essential oils present in lemongrass oils, have been reported to reduce ammonia production on incubation of mixed feed with buffalo rumen liquor [52]; however, their effects on HAB were not demonstrated. In the present study, although the growth of HAB was not affected (Table 11) either by lemongrass or clove oils (except at the highest dose), the reduction in ammonia production could be due to the modification of the diversity of HAB [53] in the enrichment culture media. However, in contrast to earlier researchers [54,55,56], no inhibition of ammonia production and growth of HAB were reported in the present study due to the addition of eucalyptus oil; this might be due to the type of bacteria isolates in the enrichment culture or dose levels used. 

*Sapindus mukorossi* fruits are a rich source of saponins [57,58] and are responsible for the decrease in rumen ammonia production, mostly by the reduction in the number of protozoa [59] and subsequent decrease in bacterial turnover; however, the results were not consistent across studies [60]. The reduction in ammonia production in the present study (Table 15) by *Sapindus mukorossi* fruit extracts in the enrichment culture of HAB might be due to changes in the permeability and some other properties of the membrane [61] of HAB, although information in these aspects is scant. *Ficus bengalensis* leaves contain a significant quantity of phenolic compounds, especially condensed tannins [62]. The reduction in protease activity by acetone extract of FB leaves has gained support from the observations of other researchers, where Molan et al. [63] in an in vitro study with condensed tannins containing the weed dock (*Rumex obtusifolius*) reported a reduced growth rate of rumen proteolytic bacteria, *C. proteoclasticum*, *B. fibrisolvens*, *S. bovis*, *Eubacterium* sp. and *P. bryantii*, and inferred that condensed tannins can modify the growth of rumen proteolytic bacteria either directly or indirectly. 

## 5. Conclusions

High-throughput sequencing of amplicons of 16S rRNA gene from metagenomic DNA recovered from an enrichment culture of HAB of buffalo rumen indicated that at the phylum level, *Proteobacteria* (61.1 to 68.2%) was the most predominant HAB followed by *Unclassified bacteria* (24–29%). At the genus level, *Candidatus Carsonella*, *Allisonella*, *Oscillobacter*, *Bacteroides*, *Proteus*, *Fusobacterium*, *Pyramidobacter*, *Paraeggerthella*, *Denitrobacterium*, *Succinoclasticum* and *Acidaminococcus* were predominant. It was also found that twenty isolates of HAB from the rumen of buffaloes had a very high rate of ammonia production (up to 200 times higher than that reported earlier) and had low similarity in 16S rRNA gene to cultured bacteria, indicating that many more culturable HAB exist than known so far. Clove oil (0.6 mL/L) and reetha (*Sapindus mukorossi*) fruit (6 mL ethanol extract/L) were found to be promising and effective in reducing the growth, protease production and the ammonia production of HAB culture. Therefore, they have future scope in use as feed additives for better utilization of proteins in animal diets as well as reduction in environmental pollution. 

## Figures and Tables

**Table 1:** Details of reaction mixture for PCR amplification of 16s rRNA gene of HAB isolates.

Reagents	Quantity
Template (Genomic DNA)	1 μL
*Primers*-F	0.25 μL
*Primers*-R	0.25 μL
10X PCR buffer	5 μL
dNTP’s (100 mM)	1 μL
MgCl_2_ (50 mM)	1.75 μL
Nuclease-free water	39.828 μL
Taq polymerase (5 U/μL)	0.25 μL
BSA (50 mg/mL)	0.672 μL
Total prepared master mixture	50 μL

**Table 2:** Details of primers used for PCR amplification of nearly full-length 16s rRNA gene of bacterial isolates.

Primer Name	Sequence (5′→3′)	Expected Amplicon Size (bp)	Annealing Temperature (°C)	Reference
27F	*AGAGTTTGATCMTGGCTCAG*	1465	55	Lane, 1991 [30]
1492R	*TACGGYTACCTTGTTACGACTT*			

**Table 3:** Major taxonomic groups of bacterial sequences recovered in enrichment culture of buffalo rumen content in medium containing casamino acid as sole energy source.

Domain	Phylum	Class	Order	Family	Genus	% of Total Bacterial Sequences			
						B1	B2	B3	B4
						100	100	100	100
Bacteria	*Proteobacteria*					61.1	68.2	67.6	61.6
,,	,,	*Gammaproteobacteria*				7.3	7.7	6.9	7.3
,,	,,	,,	Unknown	Unknown	*Candidatus* Carsonella	2.8	4.6	4.2	3.3
,,	,,	,,	*Enterobacteriales*			1	0.23	0.14	0.14
,,	,,	,,	,,	*Enterobacteriaceae*		1	0.23	0.14	0.14
,,	,,	,,	,,	,,	Proteus	0.96	0.18	0.07	0.11
,,	,,	,,	Unclassified *Gammaproteobacteria*			3.5	2.9	2.6	3.7
,,	,,	Unclassified *Proteobacteria*				53.4	60.5	60.6	54.3
,,	*Bacteroidetes*					1.8	0.94	1.2	2.1
,,	,,	*Bacteroidia*				1.1	0.75	1.2	2.1
,,	,,	,,	*Bacteroidales*			1.1	0.75	1.2	2.1
,,	,,	,,	,,	*Bacteroidaceae*		0.69	0.15	0.76	1.4
,,	,,	,,	,,	,,	*Bacteroides*	0.69	0.15	0.76	1.4
,,	*Firmicutes*					6.9	2.8	4.9	6.3
,,	,,	*Clostridia*				1.5	0.88	1.4	1.7
,,	,,	,,	*Clostridiales*			1.4	0.87	1.4	1.7
,,	,,	,,	,,	*Ruminococcaceae*		0.38	0.33	0.34	0.89
,,	,,	,,	,,	,,	*Oscillibacter*	0.35	0.16	0.27	0.78
,,	,,	*Negativicutes*				5.1	1.8	3.5	4.4
,,	,,	,,	*Selenomonadales*			5.1	1.8	3.5	0.78
,,	,,	,,	,,	*Veillonellaceae*		0.45	0.3	0.39	0.93
,,	,,	,,	,,	,,	*Allisonella*	0.43	0.26	0.15	0.73
,,	,,	,,	,,	*Acidaminococcaceae*		4.7	1.5	3.1	3.5
,,	,,	,,	,,	,,	*Acidaminococcus*	4.1	1.4	2.8	2.9
,,	,,	,,	,,	,,	*Succiniclasticum*	0.51	0.09	0.23	0.46
,,	*Actinobacteria*					1.1	0.12	0.44	0.76
,,	,,	*Actinobacteria*				1.1	0.12	0.44	0.76
,,	,,	,,	*Coriobacteriales*			1	0.12	0.43	0.75
,,	,,	,,	,,	*Coriobacteriaceae*		1	0.12	0.43	0.75
,,	,,	,,	,,	,,	*Denitrobacterium*	0.73	0.03	0.12	0.00
,,	,,	,,	,,	,,	*Paraeggerthella*	0.14	0.06	0.23	0.64
,,	*Synergistetes*					0.31	0.10	0.13	0.42
,,	,,	*Synergistia*				0.31	0.10	0.13	0.42
,,	,,	,,	*Synergistales*			0.31	0.10	0.13	0.42
,,	,,	,,	,,	*Synergistaceae*		0.31	0.10	0.13	0.42
,,	,,	,,	,,	,,	*Pyramidobacter*	0.31	0.09	0.11	0.33
,,	*Fusobacteria*					0.12	0.04	0.75	0.57
,,	,,	*Fusobacteriia*				0.12	0.04	0.75	0.57
,,	,,	,,	*Fusobacteriales*			0.12	0.04	0.75	0.57
,,	,,	,,	,,	*Fusobacteriaceae*		0.12	0.04	0.74	0.57
,,	,,	,,	,,	,,	*Fusobacterium*	0.11	0.03	0.67	0.52
,,	unclassified_Bacteria					29.1	27.6	24.8	28.1

B1 to B4 are buffalo numbers. Data for minor groups with less than 1000 reads in at least one animal are not presented.

**Table 4:** Morphology and ammonia concentration in culture medium of different isolates of HAB at different hours post-inoculation.

Particular		Ammonia Concentration (mgN/dL) *		
Isolates	Morphology	0 h	6 h	24 h
HAB 1	Cocci, Gram-positive	0.36 ± 0.12	38.0 ± 1.70	36.6 ± 3.42
HAB 2	Rod, Gram-positive	0.70 ± 0.14	17.0 ± 4.75	80.6 ± 0.46
HAB 3	Cocci, Gram-positive	0.76 ± 0.37	85.1 ± 3.73	166.0 ± 0.47
HAB 4	Cocci, Gram-negative	0.93 ± 0.45	35.6 ± 2.71	22.4 ± 1.83
HAB 5	Cocci, Gram-positive	0.53 ± 0.17	46.9 ± 3.05	30.2 ± 0.68
HAB 6	Rod, Gram-negative	0.54 ± 0.16	58.8 ± 4.07	150.2 ± 0.36
HAB 7	Rod, Gram-positive	0.30 ± 0.13	67.5 ± 3.39	166.6 ± 0.46
HAB 8	Rod, Gram-positive	0.13 ± 0.03	53.2 ± 2.37	148.2 ± 0.47
HAB 9	Rod, Gram-negative	0.40 ± 0.23	29.6 ± 2.37	90.2 ± 2.05
HAB 10	Rod, Gram-negative	0.50 ± 0.20	27.4 ± 3.73	126.8 ± 0.91
HAB 11	Rod, Gram-negative	0.90 ± 0.33	48.4 ± 3.73	161.1 ± 2.06
HAB 12	Rod, Gram-positive	0.70 ± 0.37	60.4 ± 6.78	150.8 ± 0.48
HAB 13	Rod, Gram-negative	0.40 ± 0.25	41.0 ± 0.34	33.2 ± 0.68
HAB 14	Rod, Gram-positive	0.93 ± 0.36	118.1 ± 2.04	167.8 ± 1.60
HAB 15	Rod, Gram-positive	0.67 ± 0.37	5.5 ± 0.36	3.3 ± 0.91
HAB 16	Cocci, Gram-positive	0.10 ± 0.03	42.0 ± 0.21	6.3 ± 0.74
HAB 17	Cocci, Gram-positive	0.90 ± 0.27	32.4 ± 0.68	0.9 ± 0.13
HAB 18	Rod, Gram-negative	0.27 ± 0.10	35.2 ± 0.13	3.9 ± 0.23
HAB 19	Rod, Gram-negative	0.97 ± 0.13	39.8 ± 4.07	36.4 ± 3.20
HAB 20	Rod, Gram-negative	0.70 ± 0.33	35.7 ± 0.34	39.4 ± 4.57

* Data presented as mean ± SEM.

**Table 5:** Comparative ammonia production rate in cultures of hyperammonia-producing bacterial isolates expressed per unit cell protein.

Isolates	Ammonia Production Rate (nmol NH_3_/mg Cell Protein/min) *	
	6 h	24 h
HAB 1	54,834.5 ± 1221.09	29,673.7 ± 274.45
HAB 2	238,859.6 ± 33,251.28	36,563.8 ± 207.20
HAB 3	124,372.8 ± 2724.97	50,046.8 ± 137.58
HAB 4	114,024.6 ± 4341.25	58,222.6 ± 746.35
HAB 5	191,535.2 ± 6233.89	45,731.5 ± 1035.94
HAB 6	69,219.1 ± 2397.11	31,508.1 ± 95.72
HAB 7	172,025.4 ± 4321.22	96,707.6 ± 264.94
HAB 8	64,378.2 ± 1434.78	33,584.2 ± 465.35
HAB 9	75,727.2 ± 3029.65	49,350.3 ± 1123.79
HAB 10	66,186.4 ± 4491.04	37,553.5 ± 270.39
HAB 11	74,130.2 ± 2857.36	74,859.5 ± 954.28
HAB 12	61,872.1 ± 3470.79	41,214.1 ± 497.58
HAB 13	51,450.2 ± 212.517	25,178.3 ± 518.78
HAB 14	120,893.2 ± 1040.85	64,702.6 ± 616.07
HAB 15	33,392.0 ± 1550.98	18,491.4 ± 517.98
HAB 16	245,302.6 ± 10,001.51	49,287.5 ± 215.93
HAB 17	135,931.1 ± 1420.43	6613.4 ± 283.34
HAB 18	83,671.1 ± 2000.30	10,974.7 ± 633.86
HAB 19	60,071.2 ± 3067.76	32,558.5 ± 852.59
HAB 20	97,212.9 ± 461.71	45,866.7 ± 533.85

* Data presented as mean ± SEM.

**Table 6:** Comparative protease production rate of the hyperammonia-producing bacterial isolates.

Isolates	Protease Production Rate (mIU/mg Cell Protein/mL) *	
	6 h	24 h
HAB 1	20.9 ± 1.20	49.5 ± 0.88
HAB 2	204.2 ± 4.30	27.7 ± 1.01
HAB 3	21.3 ± 0.90	18.5 ± 4.04
HAB 4	46.4 ± 1.95	158.9 ± 4.11
HAB 5	59.7 ± 1.75	92.6 ± 0.98
HAB 6	17.1 ± 0.85	12.7 ± 1.03
HAB 7	37.2 ± 0.90	35.4 ± 2.21
HAB 8	17.5 ± 0.95	13.4 ± 0.53
HAB 9	37.4 ± 1.40	33.5 ± 1.46
HAB 10	35.3 ± 1.35	18.1 ± 0.58
HAB 11	22.3 ± 0.20	28.6 ± 1.46
HAB 12	14.8 ± 1.10	16.7 ± 0.54
HAB 13	18.2 ± 0.70	46.3 ± 0.18
HAB 14	14.7 ± 0.95	23.5 ± 0.57
HAB 15	88.1 ± 3.75	343.4 ± 1.41
HAB 16	85.5 ± 0.40	476.0 ± 5.50
HAB 17	61.2 ± 2.50	431.7 ± 3.20
HAB 18	34.7 ± 1.90	170.4 ± 2.60
HAB 19	21.9 ± 1.65	54.3 ± 0.65
HAB 20	39.4 ± 3.68	70.7 ± 2.45

* Data presented as mean ± SEM.

**Table 7:** Growth rate * (absorbance values of culture medium) of top HAB isolates.

Item	0 h	6 h	12 h	24 h
HAB 3	0.03 ± 0.0015	0.14 ± 0.002	0.12 ± 0.0005	0.19 ± 0.001
HAB 6	0.03 ± 0.0015	0.15 ± 0.001	0.12 ± 0.001	0.19 ± 0.0015
HAB 7	0.02 ± 0.002	0.14 ± 0.0005	0.18 ± 0.0015	0.18 ± 0.002
HAB 8	0.02 ± 0.002	0.14 ± 0.002	0.13 ± 0.002	0.19 ± 0.0005
HAB 10	0.02 ± 0.001	0.10 ± 0.0025	0.16 ± 0.0015	0.18 ± 0.0015
HAB 11	0.03 ± 0.0015	0.14 ± 0.002	0.14 ± 0.0025	0.17 ± 0.002
HAB 12	0.02 ± 0.0005	0.15 ± 0.002	0.17 ± 0.001	0.19 ± 0.0015
HAB 14	0.03 ± 0.0005	0.16 ± 0.001	0.18 ± 0.002	0.19 ± 0.0015

* Data presented as mean ± SEM.

**Table 8:** Rate of ammonia production by the best hyperammonia-producing bacterial isolate (HAB 14) in medium containing different amino acids as sole carbon source.

Particular	Ammonia Production Rate (nmolNH_3_/mg Cell Protein/min) * at 48 h of Incubation			
	First Transfer	Second Transfer	Third Transfer	Fourth Transfer
Arg	2292.5 ± 116.62	3879.9 ± 150.47	7404.4 ± 19.70	5321.5 ± 62.69
Thr	126.9 ± 3.15	88.7 ± 1.75	614.4 ± 2.10	561.0 ± 55.16
His	1.7 ± 0.10	139.3 ± 4.50	9.8 ± 0.45	11.2 ± 4.51
Leu	1664.4 ± 54.11	69.7 ± 4.60	2066.8 ± 10.90	663.7 ± 22.57
Val	20.5 ± 1.15	60.3 ± 0.70	38.5 ± 1.45	34.6 ± 10.03
Pro	42.3 ± 1.90	15.0 ± 0.75	12.8 ± 0.15	584.3 ± 25.58
Ile	85.9 ± 1.85	37.6 ± 1.00	7.3 ± 0.20	536.8 ± 8.02
Met	23.9 ± 1.80	30.6 ± 1.00	593.4 ± 2.40	12.7 ± 10.53
Leu	1176.9 ± 33.46	971.8 ± 2.40	4175.0 ± 37.51	1563.5 ± 306.91
Control (casamino acid)	2678.0 ± 54.51	2832.0 ± 36.51	2930.0 ± 17.50	3342.0 ± 416.24

* Data presented as mean ± SEM.

**Table 9:** Rate of protease production by the best hyperammonia-producing bacterial isolate (HAB 14) on different amino acids as substrate.

Particular	Protease Production Rate (mIU/mg Cell Protein/mL) * at 48 h of Incubation			
	First Transfer	Second Transfer	Third Transfer	Fourth Transfer
Arg	44.21 ± 1.95	89.97 ± 4.74	127.76 ± 2.73	110.86 ± 1.15
Thr	57.41 ± 3.59	115.20 ± 2.59	139.34 ± 1.86	142.67 ± 1.51
His	42.94 ± 1.35	54.08 ± 1.93	75.70 ± 2.74	69.10 ± 1.94
Leu	997.68 ± 23.73	404.36 ± 4.36	430.70 ± 2.51	256.16 ± 3.30
Val	147.21 ± 3.49	351.24 ± 4.50	295.50 ± 2.47	510.60 ± 1.25
Pro	33.88 ± 1.83	174.62 ± 2.53	99.04 ± 2.86	478.83 ± 4.63
Ile	154.62 ± 4.67	220.16 ± 4.72	112.27 ± 1.86	7918.87 ± 11.61
Met	86.21 ± 3.10	357.55 ± 4.00	133.28 ± 1.82	187.75 ± 4.10
Leu	27.65 ± 1.40	51.60 ± 1.33	178.84 ± 3.24	113.05 ± 2.25
Control (casamino acid)	71.03 ± 1.29	112.96 ± 2.30	52.81 ± 1.62	110.99 ± 1.71

* Data presented as mean ± SEM.

**Table 10:** Growth rate (OD_600_) of the best hyperammonia-producing bacterial isolate on different amino acids as substrate at 48 h post-incubation.

Particular	OD_600_ 48 h of Incubation *			
	First Transfer	Second Transfer	Third Transfer	Fourth Transfer
Arg	0.25 ± 0.003	0.15 ± 0.001	0.16 ± 0.009	0.12 ± 0.008
Thr	0.19 ± 0.002	0.08 ± 0.0008	0.06 ± 0.0005	0.07 ± 0.0004
His	0.18 ± 0.002	0.13 ± 0.001	0.08 ± 0.0002	0.02 ± 0.0001
Leu	0.08 ± 0.001	0.04 ± 0.0009	0.03 ± 0.0001	0.03 ± 0.0002
Val	0.11 ± 0.003	0.05 ± 0.0008	0.05 ± 0.0001	0.04 ± 0.0001
Pro	0.14 ± 0.001	0.06 ± 0.0005	0.04 ± 0.0002	0.05 ± 0.0005
Ile	0.11 ± 0.002	0.05 ± 0.0003	0.03 ± 0.0002	0.03 ± 0.0002
Met	0.12 ± 0.004	0.04 ± 0.0001	0.04 ± 0.0003	0.02 ± 0.0001
Leu	0.19 ± 0.006	0.09 ± 0.0002	0.05 ± 0.0002	0.06 ± 0.0004
Control (casamino acid)	0.15 ± 0.003	0.38 ± 0.007	0.38 ± 0.003	0.20 ± 0.007

* Data presented as mean ± SEM.

**Table 11:** Taxonomy assignment of isolates using Ribosomal Database Project Classifier and results of 16S rRNA gene similarity search in GenBank.

Isolates	Accession Number	Taxonomy Assigned Using RDP Classifier	Nearest Taxon in Genbank (Isolates)		% Identity	Bit Score	E-Value
			Accession Number	Taxonomy			
HAB 2	PQ099813	Eggerthellaceae	KP 944195.1	*Paraeggerthella hongkongensis*	93	551 bits(610)	7e-153
HAB 3	PQ099814	Enterobacteriaceae	AB604196.1	*Escherichia coli* O124:H	99	880 bits(476)	0.0
HAB 4	PQ099815	Clostridiaceae	GU237022.1	*Escherichia coli* O111:H	99	870 bits(471)	0.0
HAB 5	PQ099816	Enterobacteriaceae	AB604196.1	*Escherichia coli* O124:H	99	880 bits(476)	0.0
HAB 7	PQ099817	Enterobacteriaceae	EF051572.1	*Clostridium botulinum* strain CDC 1656	97	401 bits(217)	3e-108
HAB 14	PQ099818	Enterobacteriaceae	GU237022.1	*Escherichia coli* O111:H-	95	741 bits(401)	0.0
HAB 18	PQ099819	Morganellaceae	KC210851.1	*Morganella morganii* strain LTC1	95	662 bits(358)	0.0
HAB 20	PQ099820	Streptococcus	LC269367.1	*Streptococcus* sp. JCM 7891	99	793 bits(429)	0.0

**Table 12:** Effects of graded doses (0.4, 0.5, 0.6 mL/L) of eucalyptus oil, lemon grass, and clove oil on ammonia production and protease concentration by mixed rumen microflora in enrichment culture.

Treatment	NH_3_-N Concentration (mgN/dL)		Protease Concentration (mIU/mL)	
	12 h	24 h	12 h	24 h
Eucalyptus oil-1	82.3 ± 0.76 ^d^	85.3 ± 0.21 ^e^	23.2 ± 0.21 ^a^	34.5 ± 0.07 ^de^
Eucalyptus oil-2	80.0 ± 2.2 ^d^	85.4 ± 0.21 ^e^	23.4 ± 0.12 ^ab^	34.2 ± 0.23 ^bc^
Eucalyptus oil-3	81.7 ± 0.56 ^d^	85.1 ± 0.37 ^e^	23.5 ± 0.12 ^abc^	34.7 ± 0.07 ^ef^
Lemon grass oil-1	67.0 ± 10.5 ^bc^	84.1 ± 1.8 ^e^	23.6 ± 0.12 ^abcd^	34.8 ± 0.01 ^ef^
Lemon grass oil-2	51.5 ± 10.7 ^bc^	78.3 ± 0.42 ^e^	23.6 ±0.12 ^abcd^	34.3 ± 0.07 ^cd^
Lemon grass oil-3	42.1 ± 4.2 ^b^	59.3 ± 1.2 ^c^	23.8 ± 0.12 ^d^	34.9 ± 0.07 ^f^
Clove oil-1	56.6 ± 2.1 ^bc^	67.8 ± 2.7 ^d^	23.4 ± 0.36 ^abc^	34.1 ±0.07 ^bc^
Clove oil-2	41.219 ± 5.5 ^b^	37.0 ± 6.0 ^b^	23.6 ± 0.21 ^cd^	33.9 ± 0.02 ^b^
Clove oil-3	23.3 ± 8.9 ^a^	13.42 ± 1.3 ^a^	23.9 ± 0.12 ^d^	33.7 ± 0.07 ^a^
Non-supplemented Control	81.0 ± 0.21 ^d^	83.4 ± 0.92 ^e^	23.7 ± 0.12 ^bcd^	34.2 ± 0.01 ^bc^
Standard error of mean (SEM)	6.96	7.39	0.102	0.125
*p*-value	<0.001	<0.001	0.011	<0.001

Mean values bearing a, b, c, d, e, f superscripts in a column vary significantly (*p* < 0.001).

**Table 13:** Effects of graded levels (0.4, 0.5, 0.6 mL/L) of eucalyptus oil, lemon grass oil and clove oil on growth rate of mixed rumen microflora in in vitro enrichment culture.

Treatment	Growth Rate	
	12 h	24 h
Eucalyptus oil-1	0.17 ± 0.009 ^cd^	0.17 ± 0.008 ^d^
Eucalyptus oil-2	0.17 ± 0.007 ^cd^	0.17 ± 0.001 ^d^
Eucalyptus oil-3	0.18 ± 0.003 ^d^	0.17 ± 0.001 ^d^
Lemon grass oil-1	0.17 ± 0.001 ^cd^	0.17 ± 0.002 ^d^
Lemon grass oil-2	0.17 ± 0.001 ^cd^	0.17 ± 0.001 ^d^
Lemon grass oil-3	0.16 ± 0.001 ^cd^	0.16 ± 0.001 ^cd^
Clove oil-1	0.15 ± 0.001 ^c^	0.15 ± 0.01 ^c^
Clove oil-2	0.12 ± 0.006 ^b^	0.13 ± 0.005 ^b^
Clove oil-3	0.08± 0.02 ^a^	0.08 ± 0.007 ^a^
Non-supplemented Control	0.17 ± 0.001 ^cd^	0.17 ± 0.001 ^d^
SEM	0.0103	0.009
*p*-value	<0.001	<0.001

Mean values bearing a, b, c, d superscripts in a column vary significantly (*p* < 0.01).

**Table 14:** Effects of graded levels (6, 12 and 18 mL/L) of plant extracts on ammonia and protease productions by mixed rumen microflora in in vitro enrichment culture.

Treatment	NH_3_-N Concentration (mgN/dL)		Protease Concentration (mIU/mL)	
	12 h	24 h	12 h	24 h
SME-1	177.5 ± 4.3 ^g^	125.3 ± 0.23 ^a^	65.1± 0.44 ^c^	44.4 ± 0.07 ^a^
SME-2	169.0 ± 0.56 ^ef^	124.9 ± 0.19 ^a^	65.9 ± 0.00 ^c^	44.3 ± 0.07 ^a^
SME-3	173.5 ± 1.8 ^fg^	123.9 ± 0.74 ^a^	65.5 ± 0.00 ^c^	43.9 ± 0.24 ^a^
SMA-1	142.4 ± 2.3 ^a^	187.1± 4.0 ^b^	47.1 ± 0.18 ^ab^	61.9 ± 0.67 ^d^
SMA-2	151.1 ± 3.1 ^b^	187.5 ± 3.0 ^b^	47.5 ± 0.14 ^b^	60.2 ± 0.70 ^cd^
SMA-3	147.3 ± 1.8 ^ab^	188.2 ± 3.0 ^b^	47.4 ± 0.01 ^b^	60.9 ± 0.01 ^cd^
FBAC-1	161.7 ± 0.45 ^d^	202.2 ± 0.96 ^de^	46.2 ± 0.92 ^a^	60.3 ± 0.44 ^cd^
FBAC-2	154.2 ± 2.5 ^bc^	197.5 ± 1.8 ^cd^	46.9 ± 0.31 ^ab^	60.6 ± 0.25 ^cd^
FBAC-3	148.9 ± 1.2 ^ab^	191.8 ± 6.1 ^bc^	47.5 ± 0.07 ^b^	61.2 ± 0.07 ^d^
FBA-1	159.1 ± 1.8 ^cd^	193.9 ± 3.0 ^bcd^	47.4 ± 0.12 ^b^	56.8 ± 1.5 ^b^
FBA-2	159.1 ± 1.5 ^cd^	200.9 ± 3.2 ^d^	47.3 ± 0.07 ^b^	59.0 ± 1.3 ^c^
FBA-3	161.6 ± 3.0 ^d^	201.6 ± 0.37 ^d^	47.6 ± 0.07 ^b^	61.3 ±0.00 ^d^
Control-A	164.2 ± 2.2 ^de^	210.1± 0.86 ^e^	47.7 ± 0.14 ^b^	60.9 ± 0.07 ^cd^
Control-E	163.56 ± 2.8 ^de^	208.23 ± 0.92 ^e^	47.2 ± 0.11 ^b^	60.2 ± 0.06 ^cd^
Control-AC	162.47 ± 3.2 ^de^	209.79 ± 0.76 ^e^	46.9 ± 0.08 ^b^	59.9 ± 0.02 ^cd^
SEM	2.95	8.70	2.17	1.95
*p*-value	<0.001	<0.001	<0.001	<0.001

Mean values bearing a, b, c, d, e, f, g superscripts in a column vary significantly (*p* < 0.01). *SME = Sapindus mukorossi* ethanol extract, SMA = *Sapindus mukorossi* aqueous extract, FBAC *= Ficus bengalensis* acetone extract, FBA = *Ficus bengalensis* aqueous extract; Control-A, E, AC are the respective control with water (A), ethanol (E) and acetone (AC).

**Table 15:** Effects of graded levels (6, 12 and 18 mL/L) of plant extracts on growth rate of mixed rumen microflora in in vitro enrichment culture.

Treatment	Growth Rate	
	12 h	24 h
SME-1	0.26 ± 0.00 ^a^	0.27 ± 0.001 ^a^
SME-2	0.26 ± 0.00 ^a^	0.26 ± 0.001 ^a^
SME-3	0.26 ± 0.001 ^c^	0.27 ± 0.001 ^a^
SMA-1	0.27 ± 0.001 ^c^	0.33 ± 0.004 ^cd^
SMA-2	0.27 ± 0.001 ^c^	0.32 ± 0.002 ^cd^
SMA-3	0.27 ± 0.00 ^d^	0.33 ± 0.003 ^cd^
FBAC-1	0.27 ± 0.001 ^c^	0.33 ± 0.001 ^cd^
FBAC-2	0.26 ± 0.00 ^b^	0.33 ± 0.002 ^cd^
FBAC-3	0.26 ± 0.001 ^b^	0.31 ± 0.003 ^b^
FBA-1	0.28 ± 0.00 ^e^	0.32 ± 0.001 ^cd^
FBA-2	0.27 ± 0.001 ^e^	0.34 ± 0.003 ^cd^
FBA-3	0.28 ± 0.001 ^e^	0.33 ± 0.002 ^cd^
Control-A	0.28 ± 0.001 ^e^	0.33 ± 0.007 ^cd^
Control-E	0.28 ± 0.001 ^e^	0.32 ± 0.002 ^cd^
Control-AC	0.27 ± 0.001 ^e^	0.32 ± 0.003 ^cd^
SEM	0.002	0.008
*p*-value	<0.001	<0.001

Mean values bearing a, b, c, d, e superscripts in a column vary significantly (*p* < 0.001). *SME = Sapindus mukorossi* ethanol extract, SMA = *Sapindus mukorossi* aqueous extract, FBAC *= Ficus bengalensis* acetone extract, FBA = *Ficus bengalensis* aqueous extract. Control-A, E, AC are the respective control with water (A), ethanol (E) and acetone (AC).

## Data Availability

The sequence datasets generated in this study have been deposited in the Sequence Read Archive of the NCBI (Bioproject accession number: PRJNA1140842). The 16S rRNA gene sequences of isolates generated in this study have been deposited in the GenBank [accession numbers (PQ099813–PQ099820)].
